# Exploring disease interrelationships in older inpatients: a single-centre, retrospective study

**DOI:** 10.3389/fpubh.2023.1110014

**Published:** 2023-06-02

**Authors:** Yiru Ma, Kang An, Keni Zhang, Han Deng, Rui Deng, Qiaoli Su

**Affiliations:** ^1^General Practice Ward/International Medical Center Ward, General Practice Medical Center, National Clinical Research Center for Geriatrics, West China Hospital, Sichuan University, Chengdu, Sichuan, China; ^2^West China School of Medicine, Sichuan University, Chengdu, Sichuan, China; ^3^Department of Internal Medicine, The Fifth People’s Hospital of Sichuan Province, Chengdu, Sichuan, China

**Keywords:** comorbidity, chronic diseases, disease visualisation, electronic medical records, older adults

## Abstract

**Background:**

Comorbidity is a common phenomenon in the older population; it causes a heavy burden on societies and individuals. However, the relevant evidence, especially in the southwestern region of China, is insufficient.

**Objectives:**

We aimed to examine current comorbidity characteristics as well as correlations among diseases in individuals aged >60 years.

**Design:**

Retrospective study.

**Methods:**

We included records of 2,995 inpatients treated at the Gerontological Department of Sichuan Geriatric Hospital from January 2018 to February 2022. The patients were divided into groups according to sex and age. Diseases were categorised based on the International Classification of Diseases and their Chinese names. We calculated the age-adjusted Charlson Comorbidity Index (ACCI), categorised diseases using the China Health and Retirement Longitudinal Study questionnaire, and visualised comorbidity using web graphs and the Apriori algorithm.

**Results:**

The ACCI was generally high, and it increased with age. There were significant differences in the frequency of all diseases across age groups, especially in individuals aged ≥90 years. The most common comorbid diseases were liver diseases, stomach or other digestive diseases, and hypertension. Strong correlations between the most common digestive diseases and hypertension were observed.

**Conclusion:**

Our findings provide insights into the current situation regarding comorbidity and the correlations among diseases in the older population. We expect our findings to inform future research directions as well as policies regarding general clinical practice and public health, especially for medical consortiums.

## 1. Introduction

There has been a dramatic increase in the ageing population in China. In the seventh population census in November 2020, individuals aged >60 years and > 65 years comprised 18.70% (264.02 million) and 13.50% (190.64 million) of the Chinese population, respectively (1). The World Health Organization reported that the Chinese older population is expected to increase from 168 million (12.4%) in 2010 to 402 million (28%) in 2040 (2).

Ageing is a risk factor for various diseases (3–6), which contributes to chronic conditions (7) that impact older populations and ultimately leads to an increase in the healthcare burden and limited economic growth (8). Several studies have elicited the impact of ageing on both public and individual levels. A study on rural families of older individuals with chronic diseases reported a cause-effect correlation between health shock and poverty (9). Another study showed that hospitalisation rates and numbers of outpatient visits are greater among individuals with senile chronic diseases (10). Moreover, older individuals with major non-communicable diseases are more prone to disabilities (11), and ageing-related chronic diseases deteriorate the overall quality of life (12).

Taken together, these negative effects of aging have led to an increased focus on chronic diseases and thereby on comorbidity, which refers to the co-occurrence of at least two diseases (13). Comorbidity is common among the older population (14), and comorbidity of chronic diseases is common overall (15, 16). Data mining of comorbidity have never been performed in Southwest of China. The China Health and Retirement Longitudinal Study (CHARLS) (17) is a national research programme for citizens of old age in China that collects high-quality data on various topics. This programme has led to the development of a questionnaire on chronic diseases, which has proven suitable and reliable. Therefore, we used the CHARLS questionnaire to categorise diseases (further details in section 2.2) and analysed comorbidity in the present study. Additionally, we compared significant differences based on sex and age groups using the age-adjusted Charlson Comorbidity Index (ACCI), which is a common tool to evaluate long-term mortality (18).

The aim of the present study was to analyse disease comorbidity, especially of chronic diseases, among older patients treated at Sichuan Geriatric Hospital. Correlations identified using a specific algorithm and differences based on sex and age groups determined using appropriate statistical methods may indicate innovative methods to study the relationship of diseases in a big data world, future research directions based on our findings, and a thinking pattern for clinical workers when treating patients with comorbidities.

## 2. Materials and methods

We collected anonymised data from the hospital information system at the Gerontological Department of Sichuan Geriatric Hospital from January 2018 to February 2022 for retrospective data visualisation. The collected data included patient age, sex, and disease diagnoses. The diagnoses were based on the International Classification of Diseases (ICD)-10 codes and Chinese names of medical diseases. We included all patients aged >60 years without other inclusion and exclusion criteria (19). The patients were divided into groups according to sex (male and female) and age (≤74 years, “younger older” persons; 75–89 years, “senior older” persons; ≥90 years, “older persons with longevity”) (17, 20). Computer-based disease categorisation was performed using the ‘subset’ and ‘sum’ functions of R version 4.2.0.^1^ Two researchers (Y.M. and H.D.) and two clinicians (K.A. and Q.S.) subsequently verified the categorisation based on the clinical code database and local clinical experiences, using the ‘find’ and ‘filter’ functions of Excel 2010 (Microsoft Office, Microsoft Corporation, Redmond, WA, United States). K.Z. and R.D. were responsible for the manual categorisation of all records. For both the ACCI and the disease categorisation of the CHARLS questionnaire, a disease condition was considered when it was included more than once in each record.

This study was approved by the Ethics Committee on Biomedical Research, West China Hospital of Sichuan University (protocol code 2019–1155), as a part of the Establishment and Evaluation of Healthcare Testing System for Older Persons program. Informed consent was not required because only anonymised data were used.

### 2.1. Age-adjusted Charlson comorbidity index

We calculated the ACCI as previously described by Charlson (21) for each age and sex group. We also used the Charlson Comorbidity Index without age adjustment (CCI) to eliminate age effects but modified several items. The scores of inpatients with hypertension were increased by 1 point, based on a recent Chinese reference (22). Diabetes was considered a 1-score item regardless of severity because our data did not provide sufficient information. Supplementary Table S1 shows the evaluation formula. Weights were assigned based on the disease; one point was assigned after each decade from the age of 40 years. For example, one and two points were assigned to the records of patients aged 50–59 and 60–69 years, respectively. We also compared the ACCI of male patients to that of female patients. All variables were non-normally distributed; they are thus presented as median and quartile values. Distributions of the ACCI or CCI according to sex or age are visualised using column stacking diagrams created in Excel 2010.

### 2.2. CHARLS

Based on the CHARLS questionnaire (23), we considered the following 14 diseases: hypertension; dyslipidaemia; hyperglycaemia or diabetes; cancer or malignant tumours; chronic lung diseases (asthma was an independent item); liver diseases; heart diseases; stroke; kidney diseases (involving the kidney but no other parts of the genitourinary system); stomach or other digestive diseases; emotional, nervous, or psychiatric problems; memory-related diseases (including Alzheimer’s disease, amnesia, dementia, Parkinson’s disease, and other diseases); arthritis or rheumatism; and asthma. The frequency and proportion of each of these diseases were calculated separately for the different sex and age groups.

### 2.3. Web graphs

**Figure 1:**
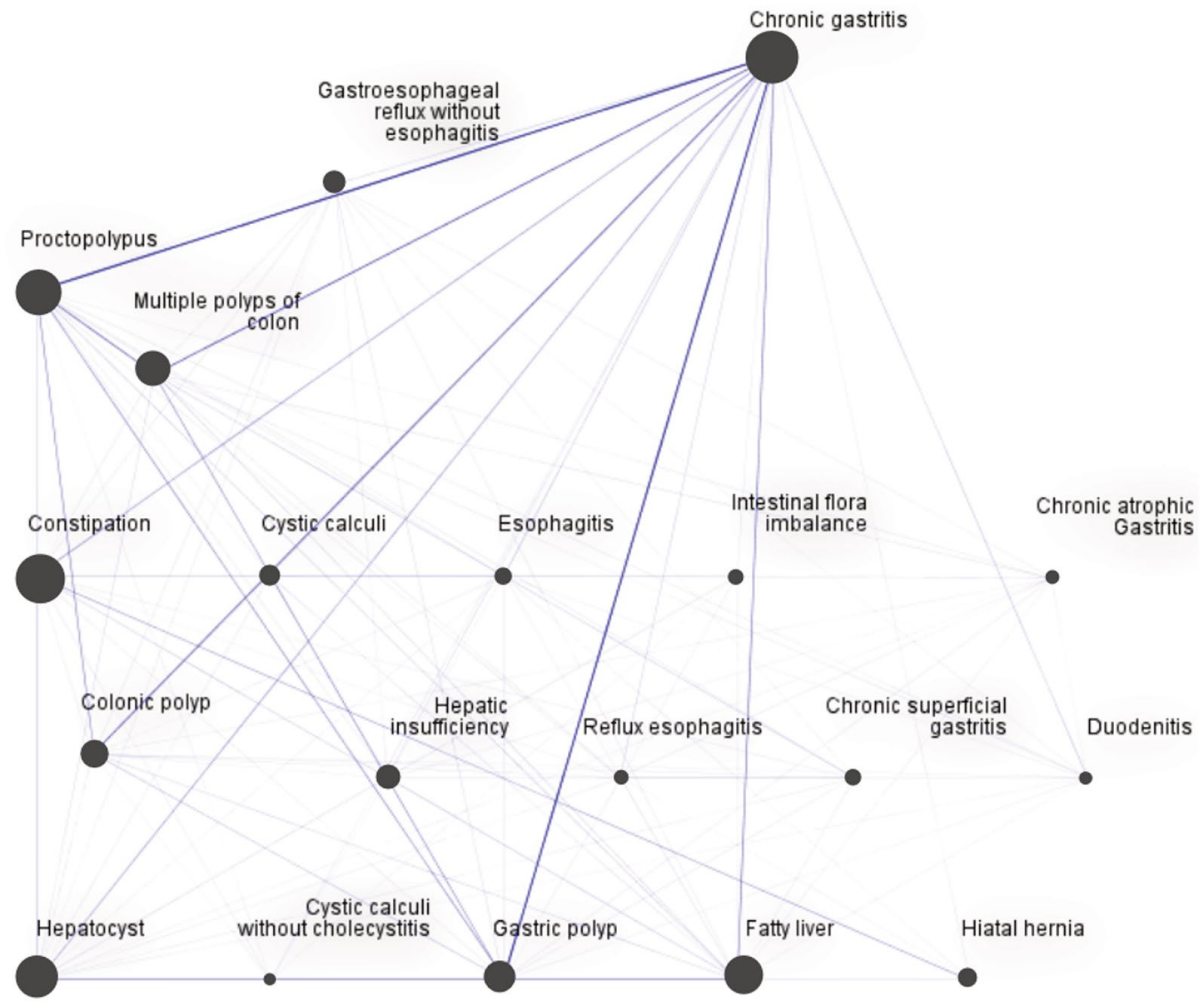
Web graph for “Liver diseases-Stomach or other digestive diseases.” This figure shows co-occurrence of diseases pairs by frequency visualization. Nodes represent diseases, and the size of nodes indicates the disease frequency. The thickness of the lines indicates the frequency of co-occurrence of the most common liver, stomach, or other digestive diseases.

Comorbidity was visualised using web graphs (24). Each node indicates a disease, and a thick line represents the comorbidity frequency of two diseases. Normal and dotted lines indicate strong/moderate and weak connections, respectively. Strong connections are defined as a comorbidity frequency of >200 cases. As an example, Figure 1 shows the web graph for ‘Liver diseases-Stomach or other digestive diseases’, where nodes represent diseases, and the difference of size between nodes indicates the rank of frequency of diseases. The thickness of the lines indicates frequency of the co-occurrence of the most common liver, stomach, or other digestive diseases.

### 2.4. Apriori algorithm

We used the Apriori algorithm to assess correlations among the 14 aforementioned diseases, especially correlations with hypertension. This is a classical method for frequent itemset mining and association discovery (25). The most crucial parameters included support, confidence, and lift. Support represents the occurrence percentage, confidence represents the conditional probability, and lift represents the dependency and correlation. Lift >1 indicates a positive connection between antecedent and consequent. The definitions are depicted below (26), where X and Y represent antecedent and consequent, respectively.


$$\begin{array}{l} \mathrm{Support}\;\left(X\to Y\right)=P\left(X\cap Y\right)\\ =\frac{numberofintersectionsconsistingofboth\;X\;and\;Y}{totalamountofcases} \end{array}$$

$$\begin{array}{l} \mathrm{Confidence}\;\left(X\to Y\right)=P\left(Y|X\right)\\ =\frac{P\left(X\cap Y\right)}{P\left(X\right)}=\frac{numberofintersectionsconsistingofboth\;X\;and\;Y}{numberofcasescontaining\;X\;} \end{array}$$

$$\mathrm{Lift}\;\left(\mathrm{X,Y}\right)=\frac{P\left(X\cap Y\right)}{P\left(X\right)P\left(Y\right)}$$

After several pre-tests, the minimum antecedent support and rule confidence were set to 0.3 and 80%, respectively. The maximum number of antecedents was set to 3.

### 2.5. Statistical analysis

Baseline characteristics and diseases according to sex or age were compared using chi-square tests (27), and the Bonferroni test was used for intergroup comparisons of disease frequency. We used the Mann–Whitney U test to compare the ACCI index according to sex and the Kruskal–Wallis H test to compare the CCI among the age groups (28). Frequency of diseases was counted using the ‘count’ function of the ‘plyr’ package of R version 4.2.0 (See Footnote 1). All statistical analyses were performed using IBM Statistical Product and Service Solutions R26.0.0.0 (IBM SPSS, IBM Corporation, Armonk, NY, United States). Statistical significance was set to *p* < 0.05, and extreme statistical significance to *p* < 0.001. IBM Statistical Product and Service Solutions Modeler 18.0.00 (IBM SPSS, IBM Corporation, Armonk, NY, USA) was used to draw web graphs and operate the Apriori algorithm.

## 3. Results

### 3.1. Participant characteristics

The 2,995 included cases consisted of 1,589 (53.1%) women and 1,406 (46.9%) men, with a significant sex-based difference in the number of cases (*p* < 0.05). The median patient age was 77.4 (± 10.7) years (range, 60–102 years). Compared with the other two age groups, the longevity group (age ≥ 90 years) showed a significantly higher number of cases (*p* < 0.001). The characteristics of all participants are summarised in Table 1.

**Table 1:** Participant baseline characteristics and Charlson Comorbidity Indexes.

	Participants	ACCI	CCI
	*N* (%)	Median (quartile)	Median (quartile)
Median age of all patients	77.4 (±10.7)	–	–
Age range	60–102	–	–
Age category	***p* < 0.001**	–	***p* < 0.001**
≤74 years old	1,291 (43.1%)^x^	4 (3–6)	2 (1–4)^a^
75–89 years old	1,204 (40.2%)^x^	7 (6–9)	4 (2–5)^b^
≥90 years old	500 (16.7%)^y^	10 (9–10)	5 (4–5)^c^
Sex	***p* = 0.001**	***p* < 0.001**	–
Female	1,589 (53.1%)	7 (5–9)	–
Male	1,406 (46.9%)	7 (5–7)	–
All records	2,995	7 (5–9)	–

Significant differences (*p* < 0.05) are indicated in bold letters. Values marked with superscript x or y were significantly different. Values marked with superscript a, b, or c were significantly different. Superscripts “*x*, *y*” and “*a*, *b*, and *c*” were not related.

ACCI, age-adjusted Charlson Comorbidity Index; CCI, Charlson Comorbidity Index without age adjustment.

### 3.2. ACCI/CCI according to age and sex

There were significant differences in the ACCI and CCI of the participants according to sex and age. Table 1 shows the medians and quartiles. Supplementary Figure S1 shows the column stacking diagrams for the ACCI according to sex. Supplementary Figures S2, S3 present the column stacking diagrams for the ACCI and CCI, respectively, according to age. The median, interquartile range, and ACCI range were 7, 5–9, and 1–23, respectively. Although the median ACCI of both sexes was 7, there was a significant difference in the distribution variation of the ACCI between the sex groups. As shown in Supplementary Figures S2, S3, age was positively correlated with both the ACCI and CCI.

### 3.3. Diseases in the CHARLS questionnaire

As shown in Table 2, the most common disease category was of diseases related to the stomach or other digestive diseases (2,101 cases), followed by hypertension (1,933 cases). We observed significant differences in the occurrence of chronic lung diseases, stroke, kidney diseases, memory diseases, and asthma according to sex. Moreover, there were significant differences in the occurrence of all diseases according to age, especially between the longevity (age ≥ 90 years) and both other groups. The details are shown in Table 3. Additionally, diseases based on the CHARLS questionnaire for deceased persons are listed in Supplementary Table S3.

**Table 2:** Diseases according to the CHARLS grouped by sex.

Diseases	Male	Female	Total	Rank	*p*
	*N* = 1,406	*N* = 1,589	*N* = 2,995		
Hypertension	928 (66.0%)	1,005 (63.3%)	1,933	2	0.116
Dyslipidaemia	356 (25.3%)	416 (26.2%)	772	8	0.591
Hyperglycaemia or diabetes	637 (45.3%)	704 (44.3%)	1,341	4	0.582
Cancer or malignant tumours	173 (12.3%)	234 (14.7%)	407	11	0.054
Chronic lung diseases	659 (46.9%)	666 (41.9%)	1,325	5	**0.006**
Liver diseases	325 (23.1%)	384 (24.2%)	709	9	0.500
Heart diseases	632 (45.0%)	728 (45.8%)	1,360	3	0.635
Stroke	544 (38.7%)	444 (27.9%)	988	6	**<0.001**
Kidney diseases	441 (31.4%)	397 (25.0%)	838	7	**<0.001**
Stomach or other digestive diseases	975 (69.4%)	1,126 (70.9%)	2,101	1	0.365
Emotional, nervous, or psychiatric problems	212 (15.1%)	236 (14.9%)	448	10	0.863
Memory-related diseases	209 (14.9%)	159 (10.0%)	368	12	**<0.001**
Arthritis or rheumatism	43 (3.1%)	55 (3.5%)	98	14	0.536
Asthma	93 (6.6%)	29 (1.8%)	122	13	**<0.001**

Significant differences (*p* < 0.05) are indicated in bold letters. Rank was based on the total count of all diseases. Proportion of frequency of each disease was calculated in the relevant sex group and is shown in parentheses.

**Table 3:** Diseases according to the CHARLS grouped by age.

Diseases	≤74 years old *N* = 1,291	75–89 years old *N* = 1,204	≥90 years old *N* = 500	Total	Rank	*p*
Hypertension	722 (55.9%)^a^	804 (66.8%)^b^	406 (81.2%)^c^	1,933	2	**<0.001**
Dyslipidaemia	434 (33.6%)^a^	295 (24.5%)^b^	43 (8.6%)^c^	772	8	**<0.001**
Hyperglycaemia or diabetes	543 (42.1%)^a^	571 (47.4%)^b^	227 (45.4%)^ab^	1,341	4	**0.025**
Cancer or malignant tumours	180 (13.9%)^a^	191 (15.9%)^a^	36 (7.2%)^b^	407	11	**<0.001**
Chronic lung diseases	386 (29.9%)^a^	543 (45.1%)^b^	396 (79.2%)^c^	1,325	5	**<0.001**
Liver diseases	365 (28.3%)^a^	300 (24.9%)^a^	43 (8.6%)^b^	709	9	**<0.001**
Heart diseases	355 (27.5%)^a^	678 (56.3%)^b^	327 (65.4%)^c^	1,360	3	**<0.001**
Stroke	221 (17.1%)^a^	457 (38.0%)^b^	310 (62.0%)^c^	988	6	**<0.001**
Kidney diseases	260 (20.1%)^a^	359 (29.8%)^b^	219 (43.8%)^c^	838	7	**<0.001**
Stomach or other digestive diseases	915 (70.9%)^ab^	815 (67.7%)^b^	370 (74.0%)^a^	2,101	1	**0.026**
Emotional, nervous, or psychiatric problems	72 (5.6%)^a^	163 (13.5%)^b^	213 (42.6%)^c^	448	10	**<0.001**
Memory-related diseases	35 (2.7%)^a^	131 (10.9%)^b^	202 (40.4%)^c^	368	12	**<0.001**
Arthritis or rheumatism	25 (1.9%)^a^	36 (3.0%)^a^	37 (7.4%)^b^	98	14	**<0.001**
Asthma	19 (1.5%)^a^	23 (1.9%)^a^	79 (15.8%)^b^	122	13	**<0.001**

Significant differences (*p* < 0.05) are indicated in bold letters. Rank was based on the total count of all diseases. Proportion of frequency of each disease was calculated in the relevant age group and is shown in parentheses. Superscripts (a, b, or c) indicate significant differences (*p* < 0.05). The superscript “ab” indicates an absence of significant difference between the values marked with superscripts “a” and “b” (*p* > 0.05).

### 3.4. Web graphs

**Figure 2:**
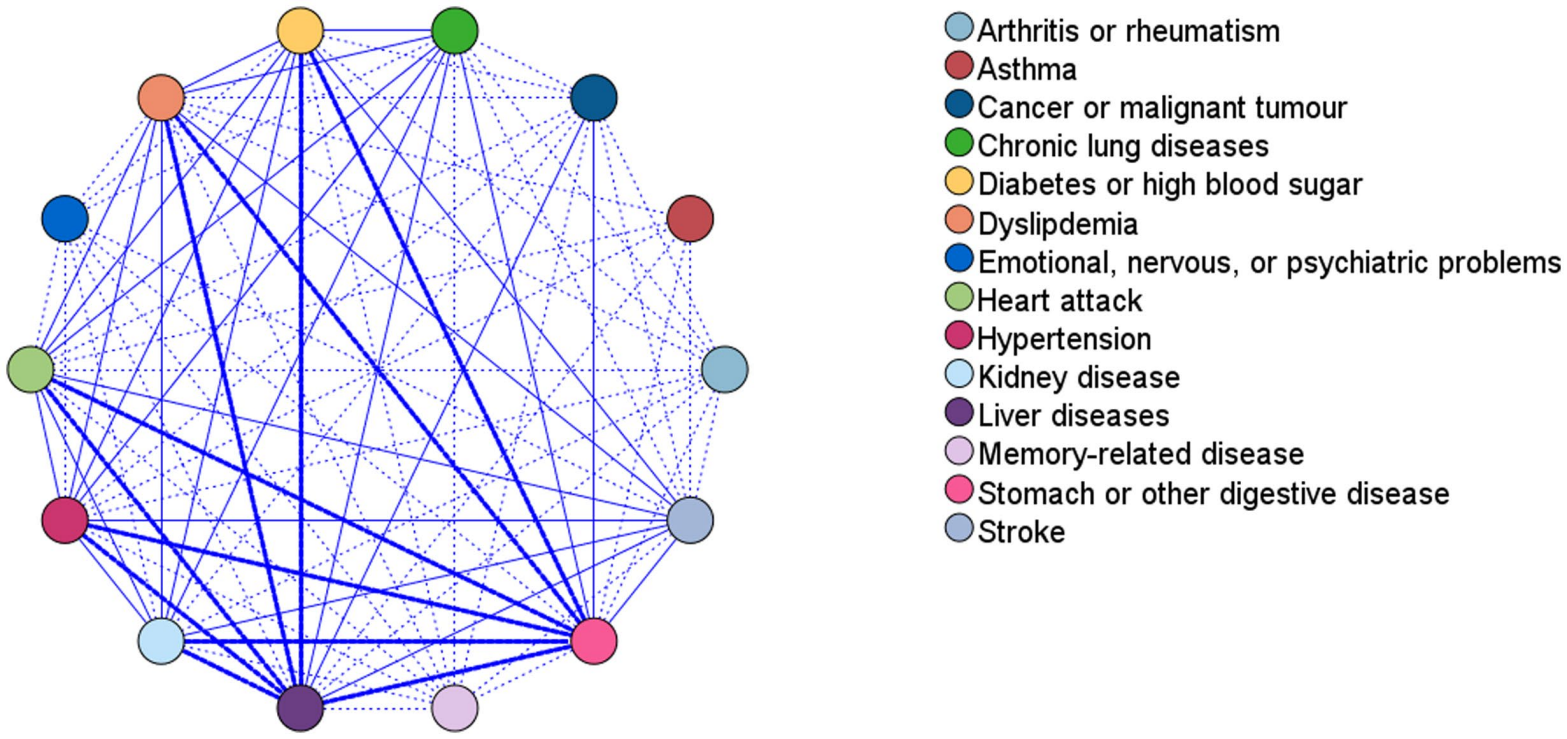
Web graph for associations between diseases. This figure shows co-occurrence of disease pairs by frequency visualization. Nodes represent diseases, thick lines show strong connection between diseases, while normal and dotted lines show medium and weak connections, respectively. Strong connections are defined as a comorbidity frequency of >200 cases. All strong connections and co-occurrence frequencies are shown in Supplementary Table S4.

Two web graphs were drawn. The first one was a general web graph. As shown in Figure 2, the pair with the strongest connection was “Liver diseases-Stomach or other digestive diseases” (709 cases), followed by “Hypertension-Stomach or other digestive diseases” (312 cases), and “Hypertension-Liver diseases” (305 cases). Supplementary Table S4 presents all strong connections. The second graph was for “Liver diseases-Stomach or other digestive diseases”. As shown in Figure 1, the pairs with the strongest connection were “Chronic gastritis-various polyps,” including “Chronic gastritis-Proctopolypus” (126 cases), “Chronic gastritis-Gastric polyp” (120 cases), “Chronic gastritis-Multiple polyps of colon” (91 cases), and “Chronic gastritis-Colonic polyp” (84 cases), followed by “Proctopolypus-Multiple polyps of colon” (82 cases) and “Chronic gastritis-fatty liver” (70 cases).

### 3.5. Apriori algorithm for association, especially for hypertension

The co-existing pairs were almost identical when the maximum number of antecedents was 2 or 3; the results were therefore stable for the top co-existing pairs, and we only analysed results for a maximum number of 3 antecedents. Hypertension was a frequent disease with a confidence of >80%. It was paired with various diseases; however, there were no remarkable combinations. We have only presented the top 10 cases for comorbidity of hypertension in a descending order (by confidence) in Table 4; more cases are shown in Supplementary Table S2. As shown in Table 4, lift was >1 for all cases, indicating a positive correlation. A higher confidence indicates more obvious coexistence between the diseases in consequent and antecedent. Additionally, as shown in Supplementary Table S2, stomach or other digestive diseases (confidence = 100%) co-existed with liver diseases alone or various combinations of liver and other diseases, which is in line with the results displayed in Figure 2.

**Table 4:** Comorbidity of hypertension in descending order by confidence (top 10).

	Antecedent	Support %	Confidence %	Lift
1	Emotional, nervous, or psychiatric problems	5.776	92.486	1.433
	Stroke			
	Chronic lung diseases			
2	Chronic lung diseases	12.521	91.733	1.421
	Diabetes or high blood sugar			
	Heart diseases			
3	Stroke	10.017	91.333	1.415
	Chronic lung diseases			
	Diabetes or high blood sugar			
4	Emotional, nervous, or psychiatric problems	6.444	88.601	1.373
	Diabetes or high blood sugar			
5	Chronic lung diseases	24.073	87.933	1.362
	Diabetes or high blood sugar			
6	Kidney diseases	7.679	87.391	1.354
	Chronic lung diseases			
	Diabetes or high blood sugar			
7	Memory-related diseases	5.743	87.209	1.351
	Emotional, nervous, or psychiatric problems			
	Stroke			
8	Kidney diseases	5.442	87.117	1.35
	Stroke			
	Diabetes or high blood sugar			
9	Stroke	16.093	86.307	1.337
	Diabetes or high blood sugar			
10	Chronic lung diseases	15.76	86.229	1.336
	Diabetes or high blood sugar			
	Stomach or other digestive diseases			

## 4. Discussion

We retrospectively visualised comorbidities among older inpatients at the Gerontological Department of Sichuan Geriatric Hospital. We observed a generally high ACCI, with differences between sex and age groups as well as correlations among the diseases.

We calculated the frequency and proportion of diseases and performed group comparisons. We used the ACCI, which is a reliable tool for predicting morbidity (29). Senior older inpatients and older individuals with longevity had high ACCI values, which is consistent with previous reports (30, 31). As shown in Table 1, both men and women had a high ACCI, which could be attributed to the fact that they were all older inpatients. Specifically, they usually present with a worse health status than that of their younger counterparts (32). Additionally, inpatients may have a heavier comorbidity burden than that in other groups. To eliminate the effect of age in our group comparisons, we used the CCI and found a positive correlation with age, which is consistent with a previous review (33). Subsequently, we calculated the frequency and proportion of diseases categorised using the CHARLS questionnaire. Some diseases did not show significant differences between the groups stratified by sex; however, there were significant intergroup differences according to age for all diseases, especially in the longevity group (age > 90 years). This age group is considered unique, and individuals of this age have been labelled ‘super-seniors’ (34, 35). However, our findings could be attributed to differences in the sample size between the older inpatients with longevity and the other older groups, with the former group comprising less than half the number of individuals in the latter groups. As for morbidity and related diseases, we found that ‘senior older’ persons had a higher morbidity rate and that digestive diseases made up for the majority of related diseases. According to the 2021 Report of Human Health Status and Key Diseases in Sichuan Province, cerebral vascular diseases, chronic obstructive pulmonary disease, ischemic heart disease, malignant tumours, and diabetes are the top 5 diseases leading to death (36). The discrepancy between our study and this official report may stem from differences in sample size. Future research is needed to investigate diseases that lead to hospitalization, especially that of older patients, on a regional basis.

We visualised the correlations between diseases using tools, such as web graphs and the Apriori algorithm (23). There were strong connections between digestive diseases, liver diseases, and hypertension; moreover, these three represent the most common diseases in our sample, which is consistent with a previous report that indicated that hypertension and digestive diseases occur frequently in this population (37). Our analysis using the Apriori algorithm yielded similar results. The correlation between liver diseases and stomach and other digestive diseases could be explained by the gut-liver axis (38) as the mutual interactions among toxins, microbes, and nutrients may influence disease development and result in comorbidity. Hypertension was correlated with various diseases; however, there were no remarkable pairs. One earlier study reported that >60 and 37% of older Chinese individuals living in rural and urban areas, respectively, presented with a comorbidity related to hypertension (39), which is consistent with the high prevalence of hypertension comorbidity in our sample. Although this could be attributed to the frailty of older individuals, there remains no evidence regarding differences in sensitivity across organs and organ systems. Additionally, we visualized the detailed comorbidities of various digestive diseases and hypertension and expect our findings to guide clinical practice and future research.

Our findings can be considered reliable, given the high-quality data and appropriate methods. First, ICD-10 codes and standardised Chinese names were recorded by clinicians and retrieved from the hospital information system by our team. Second, the combination of local clinical experience and adherence to the official ICD principles ensured the quality of disease categorisation. Additionally, manual categorisation verification by two independent researchers ensured the accuracy and reliability of the results. Third, we applied evidence-based statistics and visualisation methods. Chi-square tests are considered reliable for comparisons of proportions, unless the sample size is very small (27). Moreover, the Mann–Whitney U test and the Kruskal–Wallis H test are recommended for assessing group differences for variables with non-normal distributions (28). Web graphs were used to visualise correlations (24), while the Apriori algorithm was used for association rule mining (25).

Three points make the present study unique and ensure the generalisability of our findings. First, although similar studies have been conducted in China, few have covered the southwest of China. A previous study (40) showed that the Chinese Healthy Aging Index differed considerably between central/western China and other areas. This indicates that studies should be stratified according to geographical location. Accordingly, the present study was conducted in Chengdu, which has a high patient in- and outflow and high-quality medical resources; thus, we included an aggregated patient sample that may represent the population of southwestern China. Second, regarding the selection of study institutes, one earlier study (41) reported that specialist hospitals and tertiary hospitals were the preferred centres for >90% of patients. Specialist hospitals are widely considered better alternatives, and we therefore assume that our results are representative for the older population in southwestern China. Third, in 2017, Sichuan Geriatric Hospital joined a medical consortium led by the West China Hospital, which is an influential organisation in southwestern China. A medical consortium (41) is a China-specific model that increases healthcare efficiency by dividing tasks across various members of the medical consortium; thus, it expands the area of influence of individual institutions. Sichuan Geriatric Hospital is the only geriatric hospital in this medical consortium and was therefore a good choice for this single-centre study. To our knowledge, only a few studies based on medical consortiums have reported similar disease visualisations.

However, this study has several limitations. First, we only collected records from the Gerontology Department at Sichuan Geriatric Hospital, without considering outpatient records, other departments, other types of health institutions, and other regions, which limits the generalisability of our findings. However, our findings covered a specific time period of inpatients visiting a geriatric specialist hospital, which is a member of the southwestern medical consortium; this somewhat counterbalances the disadvantages of single-centre studies, and our findings therefore provide insights for future studies on medical consortiums. Moreover, our findings are meaningful and fill a gap in the available data, given the lack of similar studies conducted in southwestern China. Second, we did not intend to identify biological mechanisms underlying the correlations between digestive diseases and liver diseases. Future studies should include broader populations to improve the generalisability of findings and reveal biological mechanisms of comorbidity to create innovative prevention interventions and potential therapeutics using a variety of methods. Prevention interventions and therapeutics include innovative products (42), and studies on such treatment should not be restricted to quantitative methods (43).

In conclusion, this study provides novel findings that implicate changes in policies and clinical practice. We found that older individuals, especially those aged >90 years, had a higher ACCI, which predicts morbidity, and a higher proportion of diseases, highlighting the importance of implementing specific policies for older adults with longevity. The most common diseases in our patient sample were stomach or other digestive diseases and hypertension; the Chinese government is therefore expected to pay attention to the prevention of digestive diseases and hypertension, potential therapies, and other healthcare activities. As for comorbidity, we identified a strong correlation between stomach or other digestive diseases and liver diseases, while hypertension showed correlations with various diseases. Clinicians, especially general practitioners, should consider this strong relation between stomach or other digestive diseases and liver diseases, and they should emphasize hypertension as a comorbidity. These findings and their implications are very relevant for medical consortiums, especially those in southwestern China.

## Data availability statement

The original contributions presented in the study are included in the article/Supplementary material, further inquiries can be directed to the corresponding author.

## Ethics statement

The studies involving human participants were reviewed and approved by the Ethics Committee on Biomedical Research, West China Hospital of Sichuan University. Written informed consent for participation was not required for this study in accordance with the national legislation and the institutional requirements.

## Author contributions

YM was the main analyst of the data and was instrumental in the development of the programme and its methods, the analysis, and the manuscript preparation. KA was responsible for the data gathering and acted as a key member of the programme development team. HD rechecked the results and assisted in the editing of the manuscript. KZ and RD contributed to the manual disease categorisation as members of the programme development team. QS oversaw the preparation and initially developed the programme. All authors contributed to the article and approved the submitted version.

## Conflict of interest

The authors declare that the research was conducted in the absence of any commercial or financial relationships that could be construed as a potential conflict of interest.

## Publisher’s note

All claims expressed in this article are solely those of the authors and do not necessarily represent those of their affiliated organizations, or those of the publisher, the editors and the reviewers. Any product that may be evaluated in this article, or claim that may be made by its manufacturer, is not guaranteed or endorsed by the publisher.

## Footnotes

^1^ http://www.r-project.org/
