# Supplementary material for: Exploring disease interrelationships in older inpatients: a single-centre, retrospective study
Source: Front Public Health. 2023 Jun 2;11:1110014. doi: 10.3389/fpubh.2023.1110014 (PMC10272409; doi:10.3389/fpubh.2023.1110014)
Supplement: Supplementary file 1 [file Data_Sheet_1.pdf]

Supplementary Material

Exploring Disease Interrelationships in Older Inpatients: A Single-Centre, Retrospective Study

Yiru Ma*, Kang An, Keni Zhang, Han Deng, Rui Deng, Qiaoli Su*

*** Correspondence:** Qiaoli Su: 18980601358@163.com

1 Supplementary Figures and Tables

1.1 Supplementary Figures

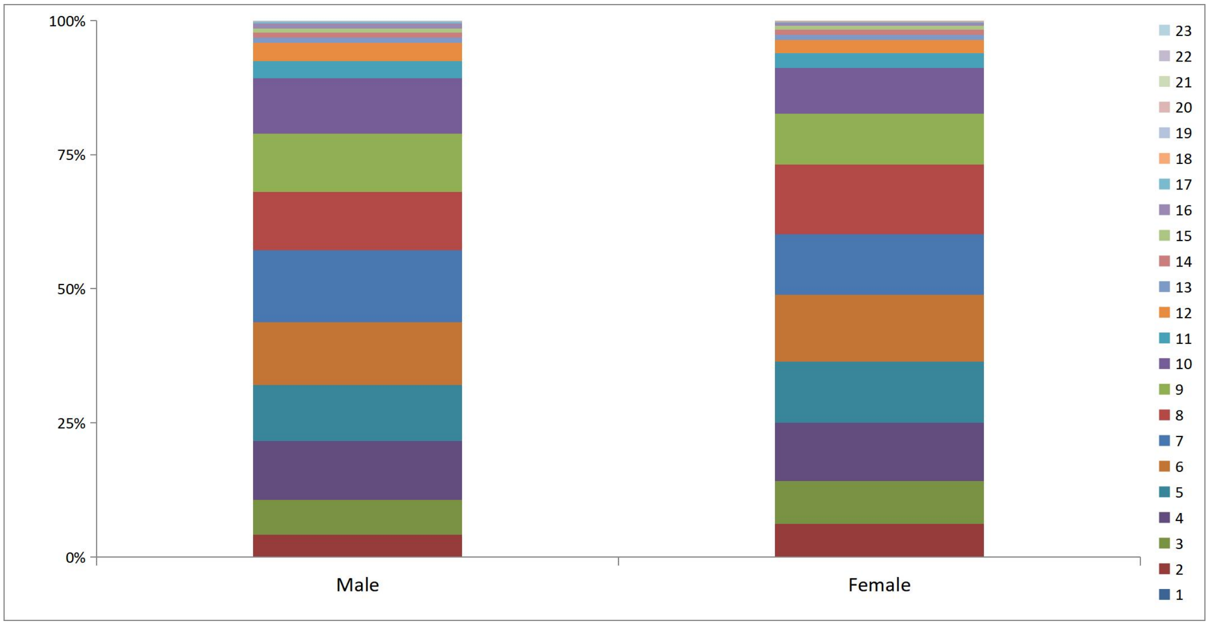

Supplementary Figure 1. ACCI across sex groups

Notes:

Abbreviations: ACCI, age-adjusted Charlson Comorbidity Index.

ACCIs (1–23 scores) marked with different colours are shown for male and female patients.

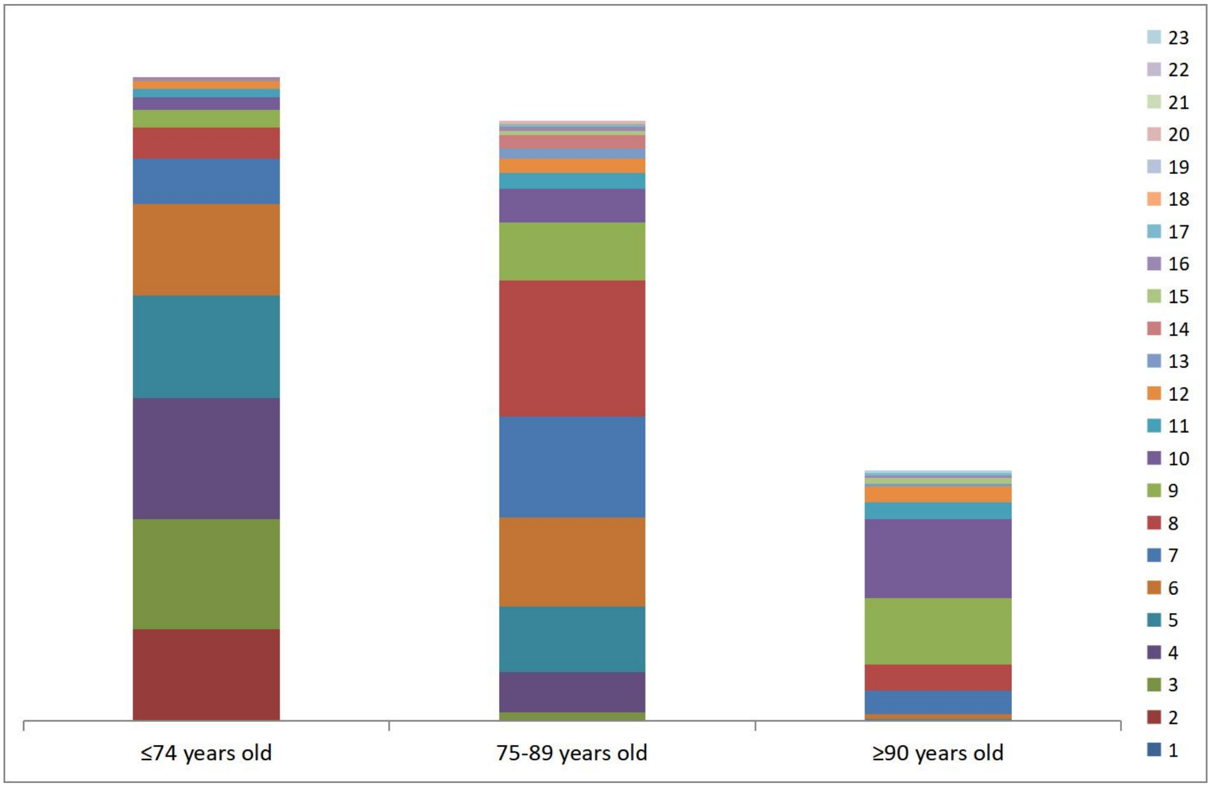

Supplementary Figure 2: ACCI across age groups

Notes:

Abbreviations: ACCI, age-adjusted Charlson Comorbidity Index.

ACCIs (1–23 scores) marked with different colours are shown for the three age groups.

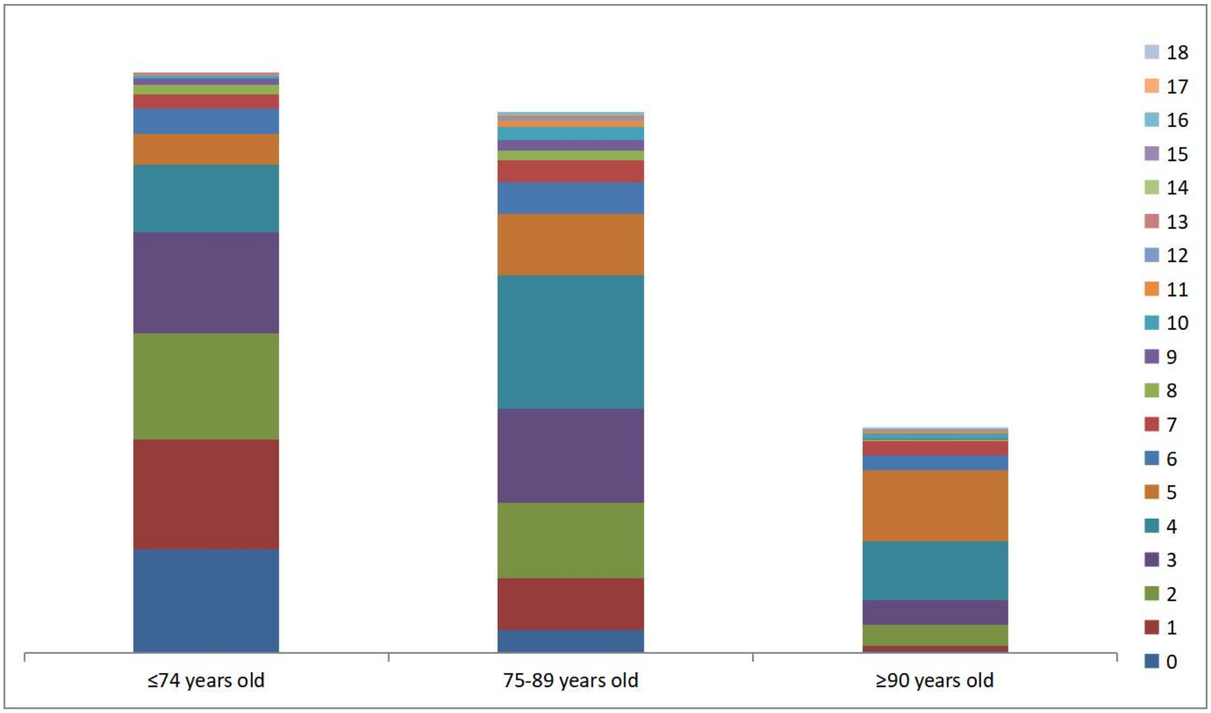

Supplementary Figure 3: CCI across age groups

Notes:

Abbreviations: CCI, Charlson Comorbidity Index without age adjustment.

CCIs (0-18 scores) marked with different colours are shown for the three age groups.

1.2 Supplementary Tables

Supplementary Table 1:

Weights assigned for specific conditions in the age-adjusted Charlson Comorbidity Index

Scores	Conditions
Assigned weights for disease	
1	Myocardial infarction
	Congestive heart failure
	Peripheral vascular disease
	Cerebrovascular disease
	Dementia
	Chronic pulmonary disease
	Connective tissue disease
	Ulcer disease
	Diabetes
	Hypertension
	Mild liver disease
2	Hemiplegia
	Moderate or severe renal disease
	Any tumour/localized solid tumour

Scores	Conditions
	Leukemia
	Lymphoma
3	Moderate to severe liver disease
6	Metastatic solid tumour
	Acquired immune deficiency syndrome(AIDS)
Assigned weights for age	
1	50–59 years old
2	60–69 years old
3	70–79 years old
4	≥80 years old

Supplementary Table 2: Disease associations identified by the Apriori algorithm

Maximum number of antecedents	Consequent	Antecedent	Support %	Confidence %	Lift
3	Stomach or other digestive diseases	Liver diseases	23.673	100	1.426
	Stomach or other digestive diseases	Liver diseases and dyslipidaemia	9.149	100	1.426
	Stomach or other digestive diseases	Liver diseases and kidney diseases	9.015	100	1.426
	Stomach or other digestive diseases	Liver diseases and stroke	6.043	100	1.426
	Stomach or other digestive diseases	Liver diseases and chronic lung diseases	5.142	100	1.426
	Stomach or other digestive diseases	Liver diseases and diabetes or high blood sugar	8.147	100	1.426
	Stomach or other digestive diseases	Liver diseases and heart diseases	9.75	100	1.426
	Stomach or other digestive diseases	Liver diseases and hypertension	10.184	100	1.426
	Hypertension	Emotional, nervous, or psychiatric problems and stroke and chronic lung	5.776	92.486	1.433

		diseases			
	Hypertension	Chronic lung diseases and diabetes or high blood sugar and heart diseases	12.521	91.733	1.421
	Hypertension	Stroke and chronic lung diseases and diabetes or high blood sugar	10.017	91.333	1.415
	Hypertension	Emotional, nervous, or psychiatric problems and diabetes or high blood sugar	6.444	88.601	1.373
	Hypertension	Chronic lung diseases and diabetes or high blood sugar	24.073	87.933	1.362
	Hypertension	Kidney diseases and chronic lung diseases and diabetes or high blood sugar	7.679	87.391	1.354
	Hypertension	Memory-related diseases and emotional, nervous, or psychiatric problems and stroke	5.743	87.209	1.351
	Hypertension	Kidney diseases and stroke and diabetes or high blood sugar	5.442	87.117	1.35
	Hypertension	Stroke and diabetes or high blood sugar	16.093	86.307	1.337
	Hypertension	Chronic lung diseases and diabetes or high blood sugar and stomach or other digestive diseases	15.76	86.229	1.336
	Hypertension	Stroke and diabetes or high blood sugar and heart	10.417	85.897	1.331

	diseases			
Hypertension	Emotional, nervous, or psychiatric problems and chronic lung diseases and stomach or other digestive diseases	6.511	85.641	1.327
Hypertension	Stroke and diabetes or high blood sugar and stomach or other digestive diseases	10.15	85.526	1.325
Hypertension	Kidney diseases and diabetes or high blood sugar and heart diseases	7.546	85.398	1.323
Hypertension	Emotional, nervous, or psychiatric problems and chronic lung diseases	9.482	85.211	1.32
Hypertension	Memory-related diseases and stroke	7.613	85.088	1.318
Hypertension	Stroke and chronic lung diseases	18.297	84.489	1.309
Hypertension	Memory-related diseases and emotional, nervous, or psychiatric problems and chronic lung diseases	5.576	84.431	1.308
Hypertension	Stroke and chronic lung diseases and stomach or other digestive diseases	11.653	84.241	1.305
Hypertension	Diabetes or high blood sugar and heart diseases	21.402	83.931	1.3
Hypertension	Stroke and chronic lung diseases and heart diseases	11.753	83.807	1.299

Hypertension	Emotional, nervous, or psychiatric problems and stroke	8.314	83.534	1.294
Hypertension	Kidney diseases and diabetes or high blood sugar	12.988	83.033	1.287
Hypertension	Emotional, nervous, or psychiatric problems and stroke and stomach or other digestive diseases	5.376	82.609	1.28
Hypertension	Diabetes or high blood sugar and heart diseases and stomach or other digestive diseases	14.324	82.051	1.271
Hypertension	Memory-related diseases and emotional, nervous, or psychiatric problems	7.913	81.857	1.268
Hypertension	Kidney diseases and stroke and chronic lung diseases	5.676	81.765	1.267
Hypertension	Cancer or malignant tumour and diabetes or high blood sugar	5.109	81.046	1.256
Stomach or other digestive diseases	Dyslipidaemia and kidney diseases	6.644	80.905	1.153
Hypertension	Memory-related diseases and chronic lung diseases	7.479	80.804	1.252
Hypertension	Kidney diseases and diabetes or high blood sugar and stomach or other digestive diseases	9.449	80.565	1.248

Hypertension	Diabetes or high blood sugar	44.775	80.537	1.248
Hypertension	Memory-related diseases and emotional, nervous, or psychiatric problems and stomach or other digestive diseases	5.175	80	1.24

Notes: Support represents the occurrence percentage, confidence represents the conditional probability, and lift represents the dependency and correlation. Lift >1 indicates a positive connection between antecedent and consequent. We set the minimum antecedent support and rule confidence to 0.3 and 80%, respectively. The maximum number of antecedents was set to 3.

Supplementary Table 3: Diseases recorded for deceased persons according to the CHARLS grouped by age

	≤74 years old	75–89 years old	≥90 years old
$N_{\text{deceased persons}}$	15	58	20
Morbidity	1.2%	4.8%	4%
Diseases		$N_{\text{diseases}}/N_{\text{persons}}$	
Hypertension	10/10	31/26	17/17
Dyslipidaemia	5/4	6/6	4/4
Hyperglycaemia or diabetes	8/6	12/12	14/9

Cancer or malignant tumours	3/3	36/36	3/3
Chronic lung diseases	3/3	28/28	18/15
Liver diseases	7/6	22/15	5/5
Heart diseases	7/7	97/32	43/14
Stroke	3/2	19/18	10/10
Kidney diseases	3/3	27/21	26/15
Stomach or other digestive diseases	21/12	82/46	26/14
Emotional, nervous, or psychiatric problems	0	4/4	3/3
Memory-related diseases	1/1	1/1	1/1
Arthritis or rheumatism	1 /1	2/2	0
Asthma	0	1/1	1/1

Notes: Morbidity = $\frac{\text{number of deceased persons in specific age group}}{\text{number of all persons in specific age group}}$; $N_{diseases}$ means number of diseases, $N_{persons}$ means number of persons with diseases.

Supplementary Table 4: Strong connections in the web graph (Figure 1)

Disease 1	Disease 2	Cases

Liver diseases	Stomach or other digestive diseases	709
Hypertension	Stomach or other digestive diseases	312
Hypertension	Liver diseases	308
Heart attack	Stomach or other digestive diseases	298
Liver diseases	Heart attack	292
Dyslipidaemia	Stomach or other digestive diseases	279
Dyslipidaemia	Liver diseases	274
Kidney diseases	Stomach or other digestive diseases	271
Liver diseases	Kidney diseases	270
Diabetes or high blood sugar	Stomach or other digestive diseases	249
Diabetes or high blood sugar	Liver diseases	244

Notes:

Frequencies of all strong connections in the web graph (Figure 1) are shown. Comorbidity frequency was calculated between disease 1 and disease 2. Strong connections were defined as a comorbidity frequency of >200 cases.
